# Intervention to improve quality of sleep of palliative patient carers in the community: protocol for a multicentre randomised controlled trial

**DOI:** 10.1186/s12912-020-00501-2

**Published:** 2020-11-16

**Authors:** Inmaculada Valero-Cantero, Yolanda Carrión-Velasco, Cristina Casals, Francisco Javier Martínez-Valero, Francisco Javier Barón-López, María Ángeles Vázquez-Sánchez

**Affiliations:** 1 Puerta Blanca Clinical Management Unit, Malaga-Guadalhorce Health District, Málaga, Spain; 2 Tiro Pichón Clinical Management Unit, Malaga-Guadalhorce Health District, Málaga, Spain; 3 MOVE-IT Research group and Department of Physical Education, Faculty of Education Sciences, University of Cadiz, Cádiz, Spain; 4 Biomedical Research and Innovation Institute of Cadiz (INiBICA) Research Unit, Puerta del Mar University Hospital, Cádiz, Spain; 5 Midlothian Foot Care, Dalkeith and National Health Service, Lothian, Scotland; 6 Department of Preventive Medicine, Public Health and Science History, Institute of Biomedical Research in Malaga (IBIMA), University of Malaga, Málaga, Spain; 7 Department of Nursing, Faculty of Health Sciences, University of Malaga, Málaga, Spain

**Keywords:** Music therapy, Caregivers, Family caregiver, Sleep, Accelerometry, Palliative care, Palliative Cancer, Home care, Oncology nursing, Nurses

## Abstract

**Background:**

Sleep disorders are commonly experienced by community caregivers for persons with cancer, with at least 72% reporting moderate to severe disorders. A consequence of this condition, which is associated with the presence of overload in the caregiver, is the increased risk of clinical depression. The aim of this study is to evaluate the effects of music on the sleep quality achieved by informal caregivers for cancer patients receiving home palliative care. In addition, we will assess the influence of specific variables that could modify these effects, analyse the correlates related to nocturnal wakefulness and consider the diurnal consequences according to the sleep characteristics identified.

**Methods:**

This single-blind, multicentre, randomised clinical trial will focus on informal providers of care for cancer patients. Two samples of 40 caregivers will be recruited. The first, intervention, group will receive seven music-based sessions. The control group will be masked with seven sessions of therapeutic education (reinforcing previous sessions). Outcomes will be evaluated using the Pittsburgh Sleep Quality Index, a triaxial accelerometer, EuroQol-5D-5L, the Caregiver Strain Index, the Epworth Sleepiness Scale and the Client Satisfaction Questionnaire. The caregivers’ satisfaction with the intervention performed will also be examined.

**Discussion:**

This study is expected to extend our understanding of the efficacy of music therapy in enhancing the sleep quality of caregivers for patients receiving home palliative care. To our knowledge, no reliable scientific investigations of this subject have previously been undertaken. Music is believed to benefit certain aspects of sleep, but this has yet to be proven and, according to a Cochrane review, high-quality research in this field is necessary. One of the main strengths of our study, which heightens the quality of the randomised clinical trial design, is the objective assessment of physical activity by accelerometry and the use of both objective and subjective measures of sleep in caregivers. Music therapy for the caregivers addressed in this study is complementary, readily applicable, provokes no harmful side effects and may produce significant benefits.

**Trial registration:**

The IMECA study is registered at Clinical Trials.gov, ClinicalTrials.gov Identifier: NCT04491110. Registered 29 July, 2020.

## Background

Restful sleep is essential to maintaining physical and mental health. Insomnia is defined as dissatisfaction with the quality of sleep, and/or the non-restorative nature of sleep, insufficient hours of sleep, frequent nocturnal interruptions of sleep and problems of sleep latency [1]. Long-term sleep disorders are associated with diverse physical, mental and neurodegenerative problems, including the development of obesity, hypertension, heart disease, diabetes and cerebrovascular accidents [2–5]. Sleep disturbance may also provoke daytime sleepiness and cause additional health-related problems [6–8].

A review of epidemiological trials reported that about one third of the general population experience symptoms of insomnia, such as difficulty in initiating or maintaining sleep. When daytime consequences are added to the definition of insomnia, the prevalence drops to 9–15%. Sleep dissatisfaction has been reported in 8–18% of the general population. Using the DSM-IV criteria for insomnia, a prevalence of 6% has been reported, with primary insomnia being most frequently cited (2–4%), followed by insomnia related to mental disorder (1–3%) [9]. Informal caregivers of persons with dependency may experience stress and dysfunctional thoughts [10]. This is often the case of caregivers for persons with cancer, receiving home palliative care. The stress is aggravated by the patient’s health situation and especially by the presence of numerous symptoms, which may change rapidly and are usually serious, requiring appropriate health care and social resources [11].

The provision of adequate home care for patients with cancer requires the presence of a main caregiver, to ensure the patient’s basic needs are met, the necessary treatment provided, the symptoms alleviated and the continuity of care maintained. The latter aspect is of fundamental importance, especially at the end of life. However, the caregiver’s physical health is often affected by these responsibilities, which can result in an excessive burden of duty [12–14] and pressure to adapt to the changes undergone by the patient [15, 16]. These circumstances often provoke a marked negative impact on the caregiver’s quality of life [17, 18]. It has been reported that the nature of sleep impairment in informal caregivers of cancer patients tends to change during the course of the disease. Thus, sleep disturbances are more prevalent among caregivers of patients with advanced cancer (42–95%) than when the patient’s cancer is at an earlier stage (36–80%) [19].

Sleep disorders are often experienced by caregivers of cancer patients in palliative care not only during the provision of care and attention [20, 21], but also after the patient’s death, and generally continue for at least the first year of bereavement [22]. One of the consequences of sleep disorder is an increased risk of clinical depression [23], which is associated with the presence of caregiver overload [24]. It is estimated that 72% of caregivers of cancer patients in home palliative care suffer moderate to severe sleep disorders, according to the Pittsburgh Sleep Quality Index, and that the duration of caregivers’ sleep (assessed by accelerometry) may be up to 44% below the recommended level [25].

Another important question, often underestimated, is that of physical activity by caregivers as a preventive health measure. The health benefits of physical activity are well known, and include a lower risk of cardiovascular disease and some forms of cancer, reduced stress and depression, greater resistance to caregiver overload, improved mental and cognitive health, physical, better sleep patterns, quality of life, and general well-being [26–29]. The WHO recommends performing at least 150 min a week of moderate aerobic physical activity, noting that physical inactivity is the fourth risk factor for mortality, worldwide [30]. Nevertheless, many caregivers do not follow this recommendation.

The current treatment approach taken to insomnia is usually pharmacological. However, the long-term use of sedative and hypnotic drugs can result in dependence and tolerance, and may reduce their efficacy [31, 32]. Furthermore, insomnia itself can produce cognitive and behavioural changes, and is associated with infrequent but severe harm [33]. In response to these considerations, non-pharmacological treatments have been developed to improve the quality of sleep [34, 35]. One such is music therapy, which is adopted in the understanding that music can arouse emotional responses, either positive or negative and of varying degrees of intensity [36].

Several theories have been advanced to explain how the brain processes emotions [37]. The classical theory focuses on the subcortical route, in which the limbic system plays a fundamental role [38]. The type of melody employed is known to influence whether a musical work is perceived as happy or sad. In the identification of melodies according to their emotional character, significant roles are played by the inferior frontal gyrus, the medial thalamus and the dorsal anterior cingulate cortex [39].

Music, as an emotional stimulus, activates different areas of the brain according to whether it is perceived as pleasant (nucleus accumbens, ‘the nucleus of pleasure’) or unpleasant (amygdala, ‘the nucleus of displeasure’) [40]. Music that produces an intensely pleasing response activates the neural reward and emotion systems in a way similar to that of other biologically significant stimuli, such as food and sex, and also as may be activated by substance abuse. This characteristic is important, as music is neither strictly necessary for survival or reproduction, nor is it a drug. The ability of music to induce such intense pleasure and to stimulate endogenous reward systems suggest that, while not essential for survival, music can greatly benefit our mental and physical well-being [41].

In 2015, a Cochrane review was conducted to assess the effects of music on insomnia in adults [42]. This meta-analysis included five studies (*N* = 264) of sleep quality, assessed by the Pittsburgh Sleep Quality Index. The review concluded that listening to music was generally beneficial. The effect size obtained suggested that sleep quality improved by approximately one standard deviation with the music-based intervention, compared to no treatment or usual treatment. Only one study (*N* = 50, low-quality evidence) reported data on sleep-onset latency, total sleep time, sleep disruption and sleep efficiency. However, this paper presented no evidence to suggest that the intervention enhanced these parameters. The authors of the meta-analysis concluded that music can be provided safely and straightforwardly and is effective in improving subjective sleep quality in adults with insomnia symptoms. However, they also observed that further research is needed to determine the effects of music on other aspects of sleep, and to take into consideration the daytime consequences of insomnia [42].

Another systematic review, performed in 2018, examined the possibility that music might improve the quality of sleep in adults with primary insomnia. These authors concluded that with respect to improving the general quality of sleep, only the relaxation associated with listening to music was statistically more effective than standard care [43]. Another study aim was to objectively assess the quality of sleep of caregivers, using an accelerometer, in addition to subjective measures, and thus determine how listening to music impacts on aspects of sleep such as sleep disruption, sleep quality, total sleep time and the daytime consequences of sleep. The accelerometer was also used to assess the physical activity of caregivers, facilitating subsequent interventions in this regard.

## Methods / design

### Aim

The aim of the study is to assess the effects of music on sleep quality in informal caregivers of cancer patients receiving home palliative care, and to evaluate the influence of specific variables. Specific aims are the following:

1. To assess the following sleep quality parameters in caregivers:
  - Sleep quality.
  - Total sleep time and rest time.
  - Sleep disruption.
  - Sleep efficiency.
2. To assess the following parameters related to wakefulness and daytime consequences, according to the sleep characteristics considered:
  - Psychological results: i) Quality of life; ii) Caregiver overload.
  - Physical results: i) Daytime sleepiness; ii) Physical activity (intensity and duration).
3. To assess the caregivers’ satisfaction with the intervention.

### Study design

The study will consist in a single-blind, multicentre, randomised controlled trial. The study will be conducted at six Primary Care Clinical Management Units belonging to the Málaga-Guadalhorce Health District (Málaga, Spain), in informal caregivers of cancer patients receiving at-home palliative care. The caregivers, will be randomised into the experimental group (EG) or the control group (CG).

### Patients and recruitment

**Fig. 1:**
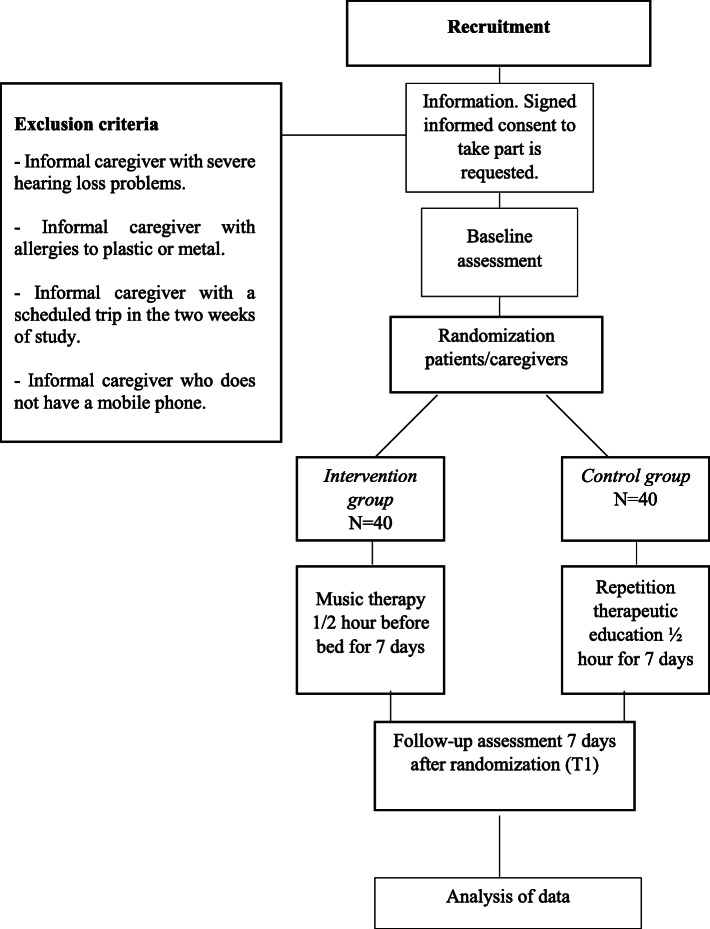
Flow chart for the Randomised Controlled Trial

The recruitment of informal caregivers will be done by using the Palliative Care Attention Process in the DIRAYA Digital Health History of the six Primary Care Clinical Management Units participating in the present study through their patients. Firstly, a selection through Epidat 4.2 program will be done, randomly selected patients and their caregivers will be contacted. Secondly, if inclusion/exclusion criteria are met, the caregivers will be fully informed of the risks, rationale, and requirements of the study, and signed informed consent will be obtained from all participants. The flow chart for the randomised controlled trial is shown in Fig. 1.

### Inclusion criteria

Inclusion criteria will include being and informal caregiver of an at-home palliative care patient, having a mobile phone and aged over 18 years.

### Exclusion criteria

Exclusion criteria will include to present impossibility of adequately hear the mp3 device or similar (severe hearing loss) or to have allergies to plastic or metal. Moreover, an exclusion criterion will be to be a formal salaried caregiver.

### Sample size

Based on the data published by Jespersen et al. 2015 [42] on sleep quality, it can be expected to find an effect equivalent to a standardized mean difference of 0.776; with an alpha error of 0.05 and a beta error of 0.10, the required sample size is two groups of 35 caregivers (Epidat 3.1), they will be increased to two groups of 40 in anticipation of possible losses.

### Control of bias

In order to avoid bias, a random assignment of the caregivers will be made to the different experimental and control conditions. Participants will not be told which group they have been assigned to, the experimental group will listen to music while controls will listen to a health education program through a mp3 device. Intention-to-treat analysis will be considered.

### Intervention protocol

#### Control group

The usual treatment will be provided as established in the Palliative Care Plan of the Andalusian Ministry of Health related to caregivers [44]. It consists of a complete initial nurse assessment of the caregivers using the 14 needs of Virginia Henderson and the subsequent care plan and monitoring of the problems or symptoms detected periodically. A basic health education will also be carried out, to improve self-care and the care of the person cared for, notions about nutrition and hydration, notions about exercise and leisure, about medication, effective communication, skin care, prevention and treatment of constipation, and notions about sleep hygiene.

The control group caregivers will receive the basic health education they received in a conventional (repeated) way, they will have to listen to it during the morning, using headphones in the mobile phone (through a shared file in Google Drive) in 30-min daily sessions during a total of 7 consecutive days. During this period, the physical activity of the caregivers will be monitored through an accelerometer.

#### Intervention group

The caregivers will receive conventional healthcare, complemented with music therapy by using pre-chosen music according to the individual preferences of participants. The music will be chosen with the assumption that “it be music that produces you personally well-being”. Participants must listen to the music 30 min before going to sleep during 7 consecutive days through headphones and mobile phone (through the Spotify Premium version program that has a free trial month). They will also have the accelerometer during 7-day intervention.

### Blinding

Masking is carried out by assigning headphones with a mobile phone and accelerometer to all caregivers in the control and intervention groups, who will be instructed not to tell the evaluator what they are listening to. Participants will not know whether they have been assigned to the control or experimental group.

### Randomisation

Caregiver will be randomised into the control or intervention group by using cards with the following sentences: “has been included in the control group” or “has been included in the intervention group”. Caregivers will not be informed about the sentence in the card that are placed in sealed opaque envelopes to ensure blinding. Participants will continue to be assigned until the pre-established sample size is reached.

The researcher responsible of the randomisation will be in charge of indicating and remembering when the caregiver puts on the accelerometer (when the nurse case manager makes the first assessment), when he/she starts listening to music or health education (1 week after the first assessment), when the use of music or health education ends, as well as the withdrawal of the accelerometer (1 week after its use) and will also be in charge of solving any doubts that arise during the study, facilitating a telephone number and email account to the caregivers participating in the study.

### Outcome assessment. Variables and measurement instruments

For the evaluation of the objectives set, a prior assessment and a final assessment will be carried out by the case management nurses of each Clinical Unit to all caregivers, both in the control and intervention groups.

The independent variables are the following:

- Condition or group: music therapy intervention program (intervention group) or conventional healthcare program (control group).
- Socio-demographic data of the caregivers: age, sex, marital status, level of studies, if they work outside the home, time dedicated to care, help received for care, time spent caring, time spent in palliative care, kinship with the person cared for, consumption of analgesic, anxiolytic and sleeping pills.

The dependent variables are the following:

- Self-perceived quality of sleep. The subjective quality of sleep will be evaluated at the beginning and at the end of the intervention with the self-completed questionnaires of the Pittsburgh Sleep Quality Index (PSQI), validated in Spanish [45]. From the score, 7 scores are obtained that inform us of as many components of sleep quality: subjective quality, sleep latency, sleep duration, “sleep efficiency”, sleep disturbances (frequency of disturbances such as coughing, snoring, heat, cold ...), use of hypnotic medication, diurnal dysfunction (ease of falling asleep while performing some activity such as fatigue). The sum of the scores obtained in each of the partial components generates a total score (TS), which can range from 0 to 21. According to Buysse et al. [46] a TS of 5 would be the cut-off point that would separate subjects who have good sleep quality from those who have poor sleep quality; a score equal to or less than 5 would indicate good sleepers.
- Assessment of the quality of sleep and physical activity objectively through accelerometry including total sleep time and rest time (night sleep and day breaks / naps), sleep disruption (number of awakenings and wakefulness after onset of sleep), and sleep efficiency (percentage of time in bed sleeping). This analysis will be performed using accelerometers for 7 days prior to the intervention week and during the intervention week, in total it will be evaluated during 14 days. Participants will wear a triaxial accelerometer (GeneActiv, Activinsights Ltd., Kimbolton, Cambs, UK, http://www.geneactiv.org/) on their non-dominant wrist 24 h a day. A sheet of instructions with questions and answers, and a contact telephone number of the person in charge of the accelerometry study, was provided in case of doubts during the evaluation period. The GeneActiv recorder contains a triaxial MEMS accelerometer with a range of ±8 g. and a sensitivity ≥0.004 g. It records both motion-related acceleration and gravitational acceleration and has linear and equal sensitivity along all three axes. This monitor provides the end user with raw data (raw or RAW) from accelerometry (ie unfiltered acceleration signals), giving the end user greater control over data processing. In this study, the accelerometer will be set to 40 Hz and 14 days will be recorded. The processing of accelerometry files is done following the following steps: 1) The file is read to collect some configuration data from the accelerometer, as well as to confirm that the reading is correct. 2) The file is processed and simplified in sections (epochs) of 5 s where the average activity measured by accelerations is collected, as well as other information that describes characteristics of the movement (such as harmonics). It is also collected at intervals of 15 min the average intensity of light that gives us an idea of whether the user is doing activities outside the house or in a dark room. This information will be taken into account, in a complementary way to accelerometry. 3) When the EPOCHS have been calculated, periods of different levels of intensity are sought (both a sedentary lifestyle and intense activities maintained continuously for most of a period of time). With them variables are created that indicate both the sedentary lifestyle such as physical activity. Phases are also located where the individual is asleep, which is reflected not only in a low activity of the accelerometer, but that it remains in a practically constant position, without changes in orientation angles. 4) The variables obtained in the previous steps are stored in a database, in addition to automatically generating a personalized PDF report for each person evaluated.
- Quality of life. The EuroQol-5D-5L questionnaire validated in Spanish [47] will be used, which describes the state of health in five dimensions (mobility, personal care, daily activities, pain / discomfort and anxiety / depression). It also consists of a visual analogue scale (VAS), graduated from 0 to 100 and with the labels “worst imaginable health status” and “best imaginable health status” on scores 0 and 100, respectively, previously used and correlated with relevant health outcomes [48, 49].
- Caregiver overload. The Caregiver Strain Index (CSI) questionnaire will be used, translated and adapted into Spanish. It is aimed at caregivers of dependent people in general. It is a semi-structured interview that consists of 13 items with a dichotomous answer True - False. Each affirmative answer scores 1. A total score of 7 or more suggests a high level of effort. This scale validated into Spanish [50] is the one used to assess the emotional and physical state in relation to caring for a person in palliative care.
- Daytime sleepiness. I was determined by using the Epworth Sleepiness Scale (ESS) [51], it is a self-administered scale of eight questions that are assessed using a scale ranging from 0 to 3, with the maximum score being 24, the scale is validated in Spanish [52].
- Satisfaction of the caregivers on the intervention carried out. It will be carried out using the Client Satisfaction Questionnaire (CSQ-8), which is self-administered and consists in 8 questions evaluated according to a 4-point Likert-type scale, with specific cut-off points for each of the items. It presents the following categories: quality of service, type of service, results and general satisfaction. The questionnaire also includes three open questions in which the patient answers about what he/she liked the most about the service, what he/she least liked and what he/she would change. This tool has demonstrated its ability to determine patient satisfaction, with high reliability and consistency, and has been validated in Spanish, in its 8-item version, and used in nursing interventions in a Spanish population [53, 54].

### Statistical analysis

Data will be expressed as mean and standard deviation for normally-distributed quantitative continuous variables or as median and interquartile range for non-normally-distributed quantitative variables and as frequencies and percentages for qualitative variables. Normality of distribution will be studied by applying the Shapiro-Wilk test and, accordingly, the change from baseline outcomes at 7 days will be compared between the control group and the intervention group by a Student’s t-test or the Wilcoxon T-test. Moreover, a multiple linear regression will be performed with the following dependent variables: sleep disruption, sleep efficiency, quality of life, caregiver overload, daytime sleepiness, physical activity, intensity and duration; and the following independent variables: the intervention, sex, age, educational level, marital status, time (hours a day) dedicated to care, if they receive care assistance, time they have been caring for, and relationship with the person they care for. The same procedure will be followed for the variables consumption of analgesics, anxiolytics and sleeping pills.

In the event that statistically significant results are obtained, in the cases of normally-distributed variables, 95% confidence intervals of the difference will be calculated to estimate between which values the difference is found. Statistical software SPSS 23.0 and Epidat 3.01 will be used. We will work with a confidence level of 95%, considering therefore, *p* values lower than 0.05 as statistically significant.

## Discussion

The expected result is to achieve improved sleep quality via an intervention based on music therapy, as a complementary treatment approach. Better sleep quality is expected to enhance caregivers’ physical and mental health, and may also decrease the consumption of medication for this purpose. This intervention is readily applicable to the usual clinical practices of caregivers providing home palliative care. It can be initiated at short notice and is totally risk-free, as corroborated in the Cochrane review “Music for insomnia in adults” [42], which confirms that the procedure is safe, easy to administer and does not interfere with other treatments or interventions.

The challenge facing this project is that of contributing to improving the health of caregivers, and the proposed means of meeting this challenge, with respect to the main caregivers of cancer patients in home palliative care, is via an innovative and economically sustainable intervention aimed at enhancing sleep quality. In addition to this direct consequence, the intervention is expected to produce a positive impact in other areas related to insomnia, such as inhibiting the development of obesity, diabetes, hypertension, heart disease, stroke, overload and stress, thus fostering immunity, reinforcing quality of life and helping prevent premature death. Insomnia also impairs carers’ daytime work performance, with prejudicial consequences for persons being treated in home palliative care. An important consideration in this respect is that palliative care in the community depends on the presence of a main caregiver; moreover, the demand for this type of care is constantly rising.

It is also important to examine caregivers’ level of physical activity, to determine whether they comply with WHO recommendations in this regard. Long-term physical inactivity is known to be one of the main risk factors for mortality, worldwide, as well as being a forerunner of frailty, sarcopenia and disability in older caregivers. These considerations would be interesting areas for further research.

The proposed study will expand our understanding of how music therapy impacts on sleep quality and on the daytime performance of informal caregivers for cancer patients in home palliative care.

## Data Availability

Not applicable; data sharing is not applicable to this article as no datasets were generated or analysed yet. The preliminary datasets generated during the study are not publicly available due it has not been completed but will be available from the corresponding author on reasonable request.

## Abbreviations

CG: Control group
CSI: Caregiver Strain Index
CSQ-8: Client Satisfaction Questionnaire
EG: Experimental group
ESS: Epworth Sleepiness Scale
EuroQol-5D-5 L: European quality of life-5 dimensions-5 levels
PSQI: Pittsburgh Sleep Quality Index
